# A Mobile App, KhunLook, to Support Thai Parents and Caregivers With Child Health Supervision: Development, Validation, and Acceptability Study

**DOI:** 10.2196/15116

**Published:** 2020-10-30

**Authors:** Rosawan Areemit, Pagakrong Lumbiganon, Chanyut Suphakunpinyo, Arunee Jetsrisuparb, Sumitr Sutra, Kunwadee Sripanidkulchai

**Affiliations:** 1 Department of Pediatrics, Faculty of Medicine Khon Kaen University Khon Kaen Thailand; 2 Department of Computer Engineering, Faculty of Engineering Chulalongkorn University Bangkok Thailand

**Keywords:** mobile app, mHealth, KhunLook, child health supervision, maternal and child health handbook, feasibility, acceptability, Thailand, mobile phone

## Abstract

**Background:**

In Thailand, children born in government hospitals receive a maternal and child health handbook (MCHH). However, when a new MCHH edition is released, those with the previous editions do not have access to the updated information. A mobile app is an appealing platform to fill this gap. We developed a mobile app called “KhunLook” as an interactive electronic MCHH intended to assist parents in child health supervision.

**Objective:**

This study describes the user requirements and development of the KhunLook mobile app, validity of parents’ growth assessments, and parents’ evaluation of feasibility and acceptability of the app.

**Methods:**

Phase 1 was a qualitative study using individual interviews. The interview data were used to revise the prototype. In phase 2, parents were randomly assigned to assess their children’s growth with the app or the MCHH. The outcomes were compared to those of the physician’s assessment, and congruence was determined. In phase 3, parents evaluated the feasibility and acceptability of the app in comparison to the MCHH through a web-based survey.

**Results:**

Four health care providers and 8 parents participated in phase 1. Two themes were identified: (1) the mobile app potentially counters parents’ infrequent use of the MCHH with accuracy, attractiveness, convenience, and simplicity, and (2) health supervision needs to be standard, up-to-date, and understandable. KhunLook was publicly launched with a family page and 7 key features: growth and nutrition, development, immunizations, oral health, reminders for the next appointment, memories, and health advice. In phase 2, 56 parents participated in the growth parameter assessments; 34 were in the App group and 22 in the MCHH group. The outcomes of the growth parameter assessments between parents and physicians in both the App and MCHH groups were not significantly different. The congruence proportions were higher in the App group for weight and head circumference, but the differences were not statistically significant. In phase 3, 356 parents from all over Thailand participated in a web-based survey. Parents rated the app feasibility as “very easy to easy” to use at higher proportions than the MCHH in all health assessment domains (growth, development, and immunizations) and ease-of-use domains with statistical significance (*P*<.001). The KhunLook app received a significantly higher mean score (8.59/10) than the MCHH (7.6/10) (*P*<.001). Most parents (317/356, 89.0%) preferred the app over MCHH. Further, 93.5% (333/356) of the parents stated that they would continue to use the app and 96.9% (345/356) would recommend others to use it.

**Conclusions:**

KhunLook, a Thai mobile app for child health supervision, was developed, validated for growth assessments, and was well accepted for ease-of-use by parents. Further studies should be conducted with a large scale of users, and the impact of this app on health behaviors and health outcomes must be evaluated.

## Introduction

Child health supervision is critical to the development of the child, family, community, and the future of populations. Periodic well-child visits foster strong relationships between the health care provider and the child and family, enabling provisioning of preventive measures such as appropriate surveillance, screening, anticipatory guidance, counseling, and immunizations [1,2]. For children, parents and primary caregivers are fundamental for the early detection of abnormalities as well as promotion of life-long practices that support their health. “Parents” in this paper refer to all primary caregivers. In Thailand, the maternal and child health handbook (MCHH) is the medium to facilitate preventive health care communication between parents and the child’s health care provider [3-5].

The MCHH was developed and provided to pregnant women at antenatal visits and for children who were born in government hospitals since 1985 [3]. It provides relevant information and serves as the standard hard copy of personal prenatal, natal, postnatal, and child health supervision record for children up to 6 years of age [3]. Only a minority of the children will use a similar book provided by hospitals in private sectors. However, health records from different sectors (ie, government hospitals, private hospitals, or clinics) are not integrated. Often, the MCHH serves as the sole continuous health supervision record.

While the MCHH is widely used and well-accepted, several studies have identified its shortcomings. The MCHH is revised intermittently to include up-to-date growth curve standards, relevant information, and changes in the country’s expanded immunization program [3]. However, parents who received previous editions do not have access to updated information, which could potentially cause conflicting messages. The MCHH has growth curves with instructions for parents to keep track of their child’s growth. Although this is practical for monitoring the health status, this part is seldomly used [3,6]. Health care providers and parents have voiced the difficulty and confusion in plotting growth curves [7-9]. Studies suggest the MCHH could be improved by including more detailed and graphical information and that it should be made from more durable material [3,10]. Printed books also carry substantial publishing and distribution costs. For the best care, parents must remember to bring the MCHH to well-child visits. Lastly, the book can be lost because of personal chaos or natural disasters. To this end, mobile health (mHealth) technology can be used to overcome the disadvantages of MCHH.

mHealth technology is defined as “medical and public health practice supported by mobile devices,” which includes delivering health care services and useful information to patients, family members, and health care providers through the use of mobile devices and communication [11]. Internet and mobile phone usage have proliferated in Thailand. The use of smartphones in Thailand increased from 26.4% in 2014 to 37.9% in 2015 and 72.3% in 2017 [11-13]. Mobile apps are conveniently distributed and downloadable to the smartphone. Parents are active users of apps. Studies have shown that parents find information on apps to support them on child health and parenting issues [14].

A mobile app is an appealing platform for the development of an interactive electronic MCHH because it addresses many of the printed book’s disadvantages [15]. Growth parameters can be automatically plotted, and percentiles can be computed and initially interpreted. Developmental milestones and immunization records can be tracked and assessed. Loss of information can be prevented through data backups. Moreover, updates in health supervision can be automatically incorporated through the app.

In this study, we developed and evaluated an interactive electronic MCHH mobile app called “KhunLook,” which translates to “my dear child” to assist Thai parents and health care providers with child health supervision. The validity of the growth assessments and user acceptability were also examined.

## Methods

### Overview

**Figure 1:**
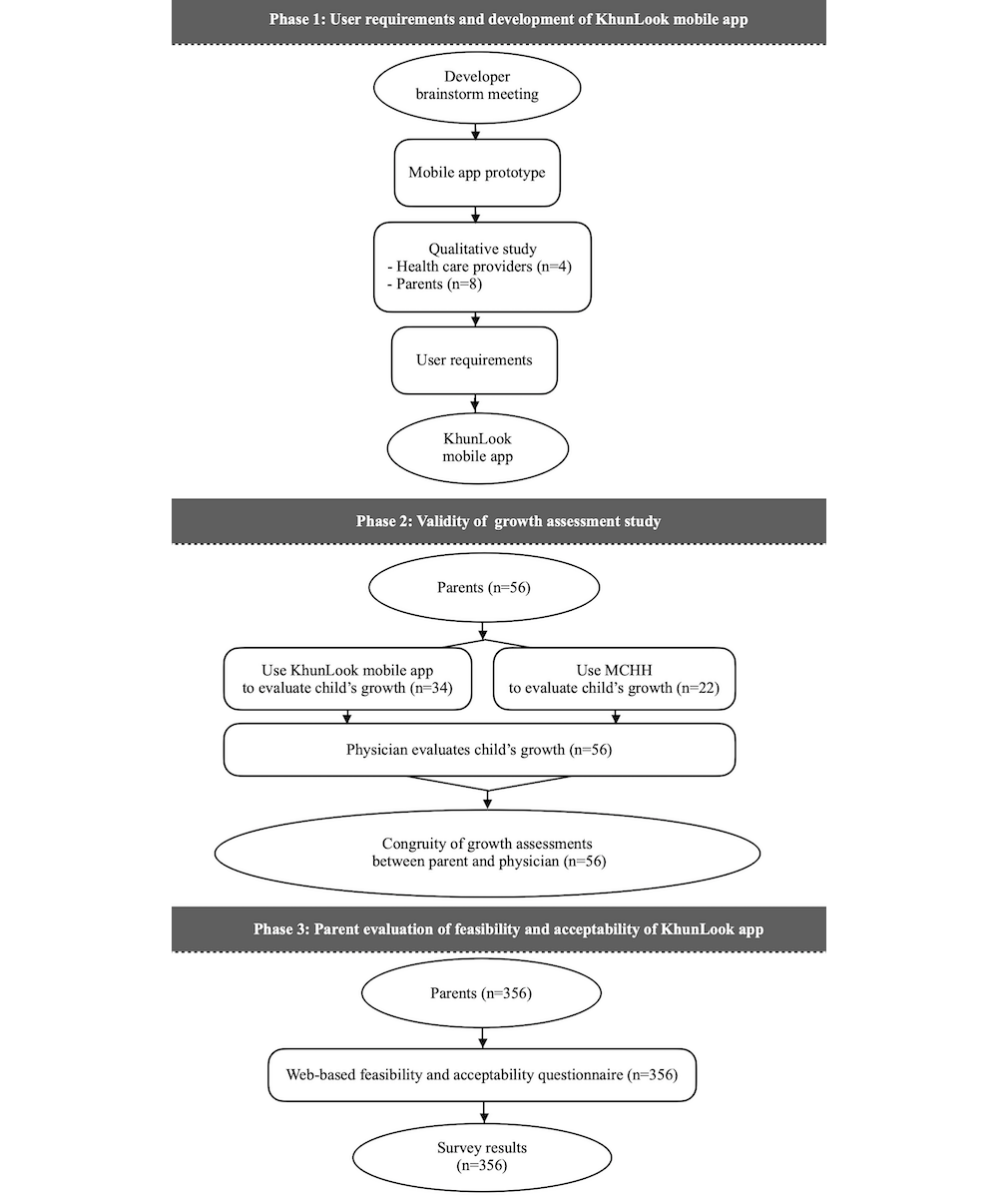
An overview of the methodology of the study. MCHH: maternal and child health handbook.

This study was approved by the Khon Kaen University Ethics Committee for Human Research. This study consisted of 3 phases: (1) phase 1, understanding user requirements and development of the KhunLook mobile app, conducted during 2013-2015, (2) phase 2, validation of the growth assessment study, conducted in 2015, and (3) phase 3, parent evaluation of the feasibility and acceptability of the KhunLook app, conducted during 2017 (Figure 1). Phases 1 and 2 were conducted at the Faculty of Medicine, Khon Kaen University, Thailand, and phase 3 was conducted through a web-based survey platform.

### Recruitment

Posters were used to publicize the study. Participation in the study was voluntary. Confidentiality was assured and written or electronic consent was obtained.

### Phase 1: User Requirements and Development of the KhunLook Mobile App

#### App Prototype

A mobile app wireframe prototype was developed based on the major requirements from a brainstorm meeting with pediatricians, dentists, and app developers (n=12). The app prototype had sections pertinent to child health supervision, including a family page, birth history, growth, development, immunizations, oral care, reminders for the next visit appointment, memories, and health advice. A panel of experts in pediatric subspecialties related to each content domain selected evidence-based and culturally appropriate content that is in accordance with the latest standards and written in a brief and comprehensible manner. The content was cross-checked for content appropriateness and understandability by 2 other subspecialists who were nationally recognized in their field, and adjustments were made until consensus was reached. The content was then incorporated into the app.

#### User Requirements

The objective was to understand user requirements in order to maximize the benefits and usage of the mobile app. The phase 1 study design was as follows. We used a qualitative design to explore participants’ perspectives by asking the question, “What are your perspectives of using a mobile app in lieu of the hard copy of MCHH?” The participants were then provided with the prototype on a tablet. They were encouraged to use and explore the prototype by themselves and then provide comments. The interviews were conducted by a trained research assistant who was not involved in the development of the app. An investigator (KS) and 2 trained research assistants who were the mobile app developers observed the process, took field notes, and assisted the participants as requested. The participants were then asked the next question, “What are your comments and additional requirements for the app?”

#### Sampling

We used purposive sampling—a nonprobability selection of participants—based on our criteria of interest for richness of data. We targeted health care professionals who worked with children and parents with children under the age of 6 years, who use smartphones and an MCHH, to obtain a wide range of perspectives [16].

#### Data Collection

We used individual in-depth interviews and field notes to collect data. Interviews were audio-recorded and transcribed verbatim, except for personal identifiers, which were removed [16]. The average health care provider interview lasted for 37.5 minutes and the average parent interview lasted for 38.8 minutes. In total, we had 7.2 hours of interview data and field notes that were analyzed.

#### Data Analysis

Data collection and analysis occurred concurrently. Researchers RA and KS read the initial 3 transcripts to develop the codes and coding categories. Codes and coding categories were discussed, compared, and clarified for the development of the initial coding schemes. After reaching consensus on the coding scheme, further transcripts were analyzed individually, followed by team discussions and consensus. Data collection continued until further themes failed to emerge. The last interview did not introduce new concepts, suggesting that theoretical saturation was achieved. After the transcripts were coded, related codes were grouped into categories and used to generate key themes. Themes and their relationships were discussed and linked to construct an understanding and address the research questions. An updated version of the KhunLook mobile app based on phase 1 input was publicly launched and made available for users to download without charge in January 2015.

### Phase 2: Validity of the Growth Assessment Study

The objectives of the phase 2 of the study were as follows: (1) to evaluate the congruence of the child growth assessments between parents and physicians and (2) to compare the proportion of congruence of parent and physician child growth assessments between the App group and the MCHH group.

#### Study Design

**Figure 2:**
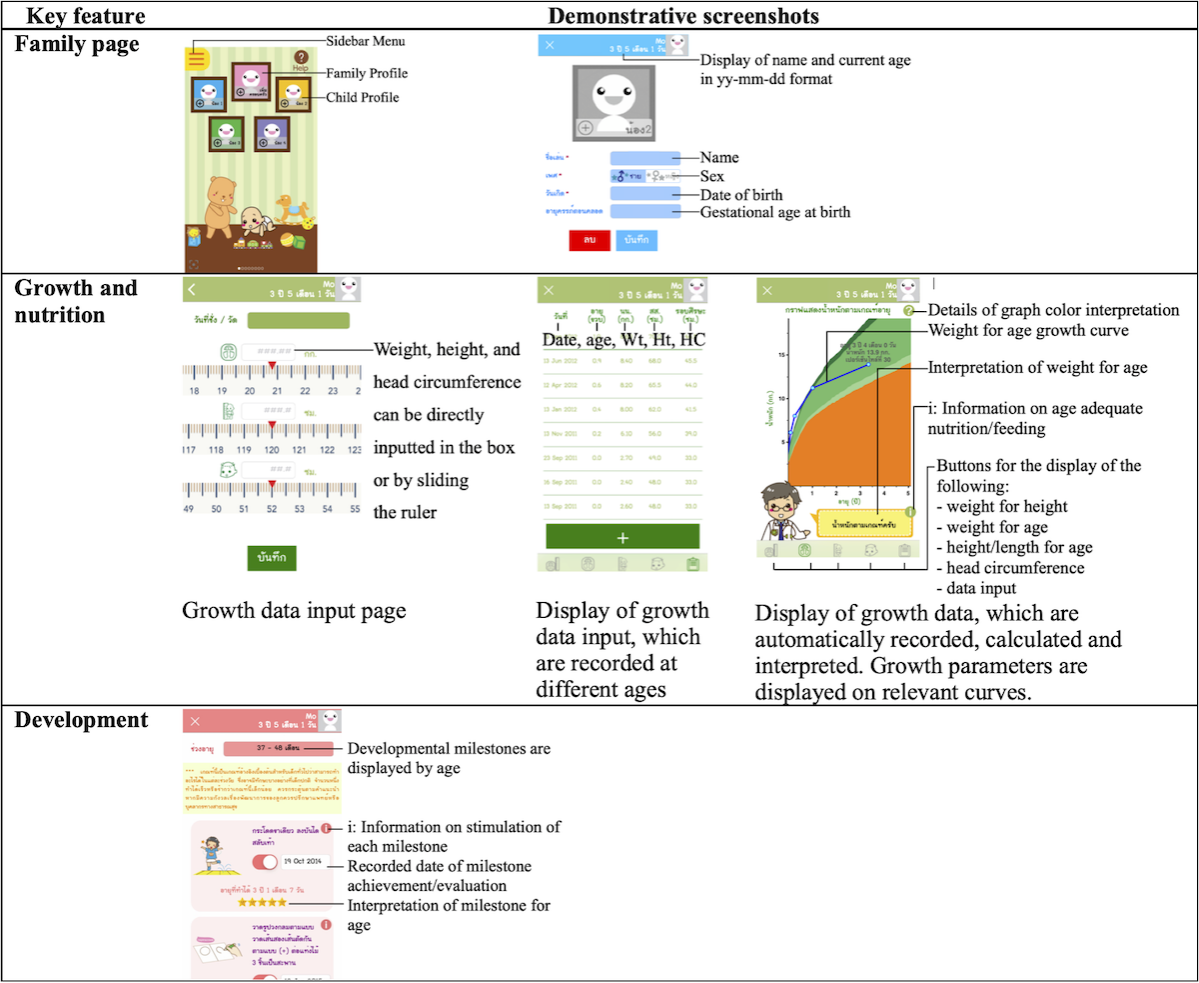
Demonstrative screenshots of the KhunLook app.

**Figure 3:**
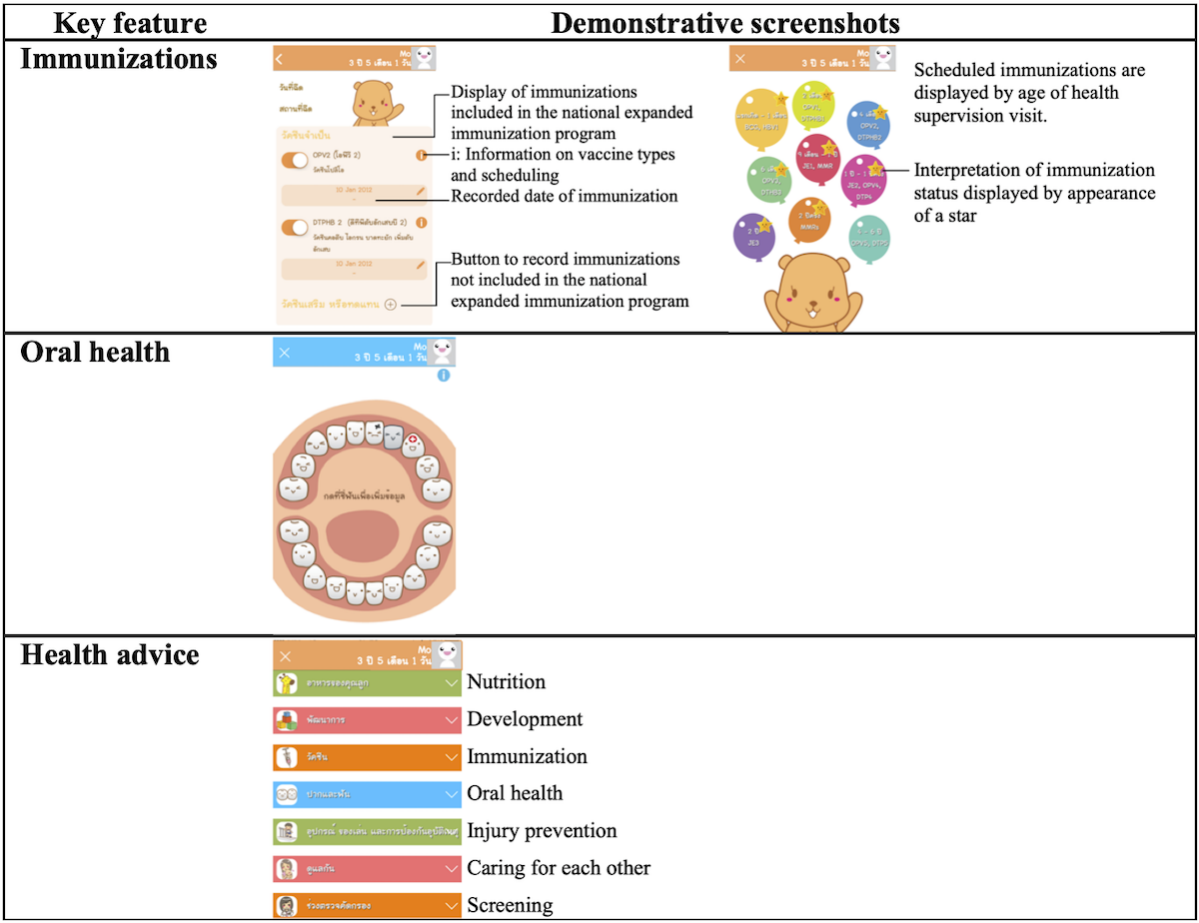
Demonstrative screenshots of the KhunLook app.

Although KhunLook has many health assessment domains as shown in Figure 2 and Figure 3, we used only the growth assessment in our phase 2 study because growth assessment is an objective, important, and practical way to monitor a child’s health. We selectively focused on this parameter to investigate whether parents could use and understand the outcomes of their child’s growth assessment. Moreover, with correct usage of the same growth curves, parent and physician growth assessments should yield the same outcomes, but with typical use, outcomes may differ. We investigated the effectiveness of the app in assisting parents in the early detection of abnormalities in their child’s growth parameters. In this quasi-experimental study design, children’s growth parameters were measured by health care providers in well-child clinics, and the parameters were provided to parents. A convenient group of parents was recruited and randomly assigned to 2 groups: the “App group” used the KhunLook app and the “MCHH group” used the MCHH to assess their child’s growth. The App group had no other guidance other than assistance with loading the app. Parents who own an MCHH are familiar with the handbook, and theoretically, app users should intuitively be able to use an engaging well-designed app without extra training [17]. The KhunLook app version 1.6 for the iPhone operating system or version 1.23 for the Android operating system, which had equivalent functionality, were used. Physicians assessed the child’s growth and provided standard child health supervision care by using the same growth chart standards. Physicians were blinded to each group. They did not use the app and they were not part of the app development team. The inclusion criteria of the parents of children under the age of 6 years were as follows: (1) own an MCHH and (2) use a smartphone/tablet with the iPhone or Android operating system.

#### Data Collection

We used a questionnaire to collect the baseline characteristics and outcomes of growth assessment by parents and physicians. Growth parameters that were assessed included weight for age, height for age, and head circumference.

#### Data Analysis

Growth parameters were categorized into 3 categories: below normal, normal, and above normal. Growth parameters were considered normal when they were between the 3rd and 97th percentile for age and sex, which was the reference provided in the app and MCHH [18]. Parents’ assessments that matched the physician’s assessment were considered congruent. For demographics, two-sided *t* test and chi-square test were used, when appropriate. The Stuart-Maxwell test for homogeneity was used to determine the agreement of the child growth assessments between parents and physicians. The chi-square test was used to determine the differences between the proportions of parent-physician agreement of child growth assessments between the App group and the MCHH group. The STATA Statistical Software (Release 14. College Station, TX: StataCorp LP) was used for the statistical calculations.

### Phase 3: Parents’ Evaluation of the Feasibility and Acceptability of the KhunLook App

The phase 3 objective was to assess parents’ evaluations of the KhunLook app in terms of feasibility and acceptability.

#### Study Design

For a wider range of user evaluations, a web-based survey was used to collect the parents’ evaluations of the KhunLook app and the MCHH in comparable sections in terms of feasibility and acceptability. The survey was launched in July 2017. The inclusion criteria of the parents of children under the age of 6 years were as follows: (1) own an MCHH and (2) used the KhunLook app within the past month.

#### Data Collection

We used a web-based questionnaire to collect information on the baseline characteristics, feasibility, and acceptability of the app and MCHH. For feasibility, parents rated the ease of using the app compared to the MCHH on 5 health assessment domains and 6 ease-of-use domains on a 4-rank rating scale (very easy, easy, difficult, and very difficult). For acceptability, parents were asked to provide an overall score from 0 to 10 (0=least acceptable, 10=most acceptable) for the app and MCHH; they were asked to choose the preferred method, if they would continue to use the app, and if they would advise others to use the app.

#### Data Analysis

For feasibility, the first 2 ratings were grouped as “very easy to easy” and the last 2 were grouped as “difficult to very difficult,” and then, the proportions were calculated. We used McNemar test for change to compare the feasibility ratings between the mobile app and MCHH. For acceptability, a paired two-sided *t* test was used to compare the scores between the KhunLook app and MCHH. The STATA Statistical Software was used for the statistical calculations.

## Results

### Phase 1: User Requirements and Development of the KhunLook Mobile App

#### User Requirements

Four health care providers and 8 parents participated in this phase. Half of the health care providers were women with a mean (SD) age of 49 (19) years (range, 36-76 years) and 50% (2/4) had practice experiences of more than 15 years. Three providers were pediatricians and one was a dentist. All of the parents were mothers with mean (SD) age of 32 (2.7) years (range, 30-36 years), and regarding their levels of education, 75% (6/8) had a doctorate degree and 25% (2/8) had a bachelor’s degree; 88% (7/8) of the mothers had a monthly income of more than 15,000 Baht per month. Five mothers had 2 children, while 3 had 1 child. The mean (SD) age of their children was 3.6 (2.3) years (range 0.9-6 years). Five mothers used iPhones and 3 used Android smartphones. One mother had previously used mobile apps to track her child’s health. From the qualitative data analysis, we identified 2 major themes that were raised by the participants and relevant to our research questions. These themes are discussed in greater detail along with the representative quotes below (Table 1).

**Table 1:** Themes and the illustrative quotes.

Theme	Illustrative quote
The mobile app potentially counters parents’ infrequent use of the MCHH^a^ with accuracy, attractiveness, convenience, and simplicity.	…*I don't know how many parents even open the MCHH. Most of them don't. They tell me it's for the doctor to read even when previous versions have included a section for parents to record. The MCHH changes from year to year and parents sometime forget to bring it to health supervision visits. Some lose them in floods. An app could easily and accurately help them check if their numbers (child’s growth parameters) are normal, and it should provide brief information for further actions*. [Quote 1, Health care provider 2] …*When I had my first child, I always read the MCHH and plotted his growth parameters. As for my second child, I just used the MCHH to record vaccines. I rely on the doctors for immunization appointments. I never recorded immunizations either; the doctor does that. I don’t really record things in the MCHH book. I bring it along for health supervision visits, not sick visits. If there were an app on my mobile, it would make things more convenient.* [Quote 2, Parent 1] …*This is great! But does it need to be constantly connected to the internet? Because not many people will pay for the charge, parents in remote areas won’t.* [Quote 3, Parent 2]
Health supervision needs to be standard, up-to-date, and understandable.	…*For child health supervision, the app needs to have sections about growth, vaccination, development, which are standard and up-to-date. Once guidelines or standards change, they should be reflected in the app. The MCHH cannot do this. The parameters should be corrected for premature babies.* [Quote 4, Health care provider 3] *I don’t think the parents understand what the Bacillus Calmette-Guérin vaccine is; it needs to be explained in Thai briefly. I think all vaccinations should be presented by age; that makes it easier for parents.* [Quote 5, Health care provider 4] …*I would use it for growth and development assessment; it should tell the parent what to do if their child has abnormal development, like go see the doctor or the kind of stimulation that is needed, and it should use a corrected age for preterm babies. I’ve searched for apps that could do this but have not yet found any that are free of charge.* [Quote 6, Parent 3] *...This app is very complete, organized, and easy to use. I can automatically assess my child’s growth, development, vaccines. The information comes from a reliable source and it even has a journal where I can add pictures. I would definitely use this app.* [Quote 7, Parent 4]

^a^MCHH: maternal and child health handbook.

Theme 1: Health care providers and parents expressed that mobile apps are potentially useful by countering the infrequent usage of the MCHH and by helping caregivers to understand their child’s health. The downside would be the need for internet connectivity.

Theme 2: Health care providers and parents expressed the need for standard and up-to-date information that is in Thai language.

#### KhunLook Mobile App

The updated version of the KhunLook mobile app based on phase 1 input contains a family page and 7 key features: growth and nutrition, development, immunizations, oral health, reminders for the next visit appointment, memories, and health advice. Demonstrative screenshots are shown in Figure 2 and Figure 3. For assessments of growth, development and immunizations, parents are required to input data for the app to record and automatically calculate, interpret, and display.

Growth standards were based on the Thai national growth reference [18], and growth parameters were assessed in relation to sex and chronological age for term infants (born at gestational age≥37 weeks) and in relation to corrected age (up to gestational age of 40 weeks) until 2 years of age for preterm infants born at gestational age <37 weeks. Assessment of development was based on the Royal College of Pediatricians of Thailand’s child developmental milestone reference [19]. Immunization status was assessed based on the Ministry of Public Health and the Pediatric Infectious Disease Society of Thailand’s immunization schedule standard [20,21]. Health advice was based on the Ministry of Public Health and the Royal College of Pediatricians of Thailand child health supervision guidelines [22]. After installation, the app fully functions without requiring internet connectivity, as mobile broadband internet use was not prevalent in Thailand when the app was first launched. Data inputted by users were stored locally on the mobile phone to ensure user privacy. The overall design of the app is colorful and playful to appeal to the target user group. Internet connection is only required for app updates and optional data backup.

### Phase 2: Validity of the Growth Assessment Study

Of the 56 parents who participated, 34 were in the App group and 22 were in the MCHH group. The baseline characteristics of the parents are shown in Table 2. There were no statistically significant differences between the parental characteristics in both the groups. The differences in the children’s mean age was significantly less, and there was a lower proportion of girls in the App group. The distribution of the growth parameters was similar in both the groups. Most of the children received an MCHH version printed within 1 year of birth (36/56, 64%), and some received versions printed within 1-2 years (12/56, 22%) and >3 years (8/56, 14%) of birth. The congruence of the child growth assessments between parents and physicians are shown in Table 3. There were no statistically significant differences between the outcomes of the growth parameter assessments between parents and physicians in both the App and MCHH groups. The proportions of congruence in the App group for weight and head circumference were higher than those in the MCHH group but the differences between each group were not statistically significant (*P*=.65, *P*=.54, and *P*=.13 for weight for age, height for age, and head circumference between the App and MCHH group, respectively).

**Table 2:** Baseline characteristics of the parents and the children by group.

Demographics				App group, n=34	MCHH^a^ group, n=22	*P* value	
**Parents**							
	Sex, female, n (%)			27 (79)	17 (77)	.85	
	**Age (years)**						.81
		Mean (SD)		33.7 (5.4)	34.0 (5.8)		
		Range		20-45	21-44		
	**Education, n (%)**						.11
		Less than bachelor’s degree		3 (9)	5 (23)		
		Bachelor’s degree		12 (35)	11 (50)		
		Master’s or doctoral degree		19 (56)	6 (27)		
	Monthly income <15000 Baht/month^b^, n (%)			3 (9)	3 (14)	.65	
	**Type of phone, n (%)**					.12	
		iPhone		24 (71)	11 (50)		
		Android		10 (29)	11 (50)		
	Parents who used other apps to assess child’s health in the past, n (%)			8 (24)	7 (3)	.49	
**Children**							
	Sex, female, n (%)			11 (32)	13 (59)	.048^c^	
	**Age (years)**						.002^c^
		Mean (SD)		2.6 (1.3)	3.8 (1.6)		
		Range		0.3-4.9	0.1-6		
	**Growth parameters,** **n (%)**						
		**Weight**				.82	
			Below normal	1 (3)	1 (5)		
			Normal	31 (91)	19 (86)		
			Above normal	2 (6)	2 (9)		
		**Length/height**				.90	
			Below normal	1 (3)	1 (5)		
			Normal	32 (94)	20 (90)		
			Above normal	1 (3)	1 (5)		
		**Head circumference**				.75	
			Below normal	0 (0)	0 (0)		
			Normal	33 (97)	21 (95)		
			Above normal	1 (3)	1 (5)		
**MCHH version**							
	Year (range)			2005-2014	2007-2013		

^a^MCHH: maternal and child health handbook.

^b^1 USD=35.3 Baht.

^c^Differences were statistically significant at *P*<.05.

**Table 3:** Congruence of child growth assessments between parents and physicians by group.

Group, growth parameter assessment		Parent, normal assessment, n (%)		Physician, normal assessment, n (%)		Congruence of assessment outcome, n (%)		*P* value	
**App group (n=34)**									
	Weight		29 (85)		31 (91)		31 (91)		.16
	Length/height		29 (85)		32 (94)		31 (91)		.22
	Head circumference		32 (94)		33 (97)		33 (97)		.32
**MCHH^a^ group (n=22)**									
	Weight		17 (77)		19 (86)		20 (91)		.37
	Length/height		20 (91)		20 (91)		21 (95)		.61
	Head circumference		18 (82)		21 (95)		19 (86)		.08

^a^MCHH: maternal and child health handbook.

### Phase 3: Parents’ Evaluation of the KhunLook App

In this study, 356 parents from all regions of Thailand participated in the web-based survey. The baseline characteristics are shown in Table 4. Evaluation of the KhunLook app compared to the MCHH for feasibility in terms of health assessment and usage are shown in Table 5. Parents rated the feasibility of the app as “very easy to easy” to use at higher proportions than the MCHH in all domains with statistical significance (*P*<.001, Table 5). The majority (354/356, 99.4%) rated the installation of the KhunLook app as very easy to easy.

**Table 4:** Baseline characteristics of the parents and children in the web-based survey (n=356).

Demographics				Values
**Parents, n=356**				
	Sex, female, n (%)		331 (93.0)	
	**Age (years)**			
		Mean (SD)	28.1 (6.1)	
		Range (years)	18-45	
	**Region of Thailand, n (%)**			
		North	50 (14.0)	
		Northeast	57 (16.0)	
		East	54 (15.2)	
		Central	147 (41.3)	
		South	25 (7.0)	
		West	23 (6.5)	
	**Education, n (%)**			
		Less than bachelor’s degree	31 (8.7)	
		Bachelor’s degree	217 (61.0)	
		Master’s or doctoral degree	108 (30.3)	
	Monthly income < 15,000 Baht/month^a^, n (%)		25 (7.0)	
	**Number of children, n (%)**			
		1	281 (78.9)	
		2	59 (16.6)	
		>3	16 (4.5)	
	**Phone operating system, n (%)**			
		iPhone	229 (64.3)	
		Android	127 (35.7)	
	Parents who used other apps to assess child’s health in the past, n (%)		24 (6.7)	
**Children, n=356**				
	Age (years), mean (SD)		2.92 (2.9)	
	Sex, female, n (%)		131 (36.7)	

^a^1 USD=33 Baht.

**Table 5:** Parents’ ratings of feasibility: health assessment and convenience of use of mobile app versus maternal and child health handbook (n=356).

Feasibility		App	MCHH^a^	*P* value
**Health assessment (very easy to easy), n (%)**				
	Weight	352 (98.9)	268 (75.3)	<.001
	Length/height	350 (98.3)	268 (75.3)	<.001
	Head circumference	350 (98.3)	271 (76.1)	<.001
	Development	353 (99.2)	279 (78.4)	<.001
	Immunization	333 (93.5)	284 (79.8)	<.001
**Convenience of use (easy to easy), n (%)**				
	Data input	352 (98.9)	287 (80.6)	<.001
	Access to desired segment	340 (95.5)	248 (69.7)	<.001
	Understandability of content	342 (96.1)	290 (81.7)	<.001
	Applicability of content	346 (97.2)	282 (79.2)	<.001
	Usefulness	354 (99.4)	261 (73.3)	<.001
	Overall convenience	354 (99.4)	252 (70.8)	<.001

^a^MCHH: maternal and child health handbook.

For acceptability, on a scale of 10, the KhunLook app received a mean (SD) score of 8.59 (1.1) (range 3-10), which was significantly higher than that of the MCHH (7.6 [1.8], range 0-10; *P*<.001). The KhunLook app received a higher score than the MCHH from 199 (55.9%) parents, equal scores from 138 (38.7%) parents, and lower scores from 20 (5.6%) parents of the total population of 356 parents. If parents had to choose a between the app and MCHH, most (317/356, 89.0%) preferred to use the app. In addition, 93.5%, (333/356) of parents stated that they would continue to use the app and 96.9% (345/356) stated that they would recommend others to use it.

## Discussion

### Principal Results

This is the first study to describe the development and evaluation of a mobile app for child health supervision by comparing the congruence of parent-physician growth assessments and parental evaluation in Thailand. Our participants in phase 1 offered insight into the requirements of an ideal app. Participants suggested that the app could counter parents’ infrequent use of the MCHH book. They also emphasized the importance of standard, up-to-date, understandable content, and offline functionality. Parents who used the app or the MCHH could comparably assess their child’s growth status to that of a physician’s assessment. Parents who used the app had higher proportions of congruence to the physician’s assessment for weight and head circumference than parents who used the MCHH, but the differences were not statistically significant. For acceptability, parents rated the app significantly more feasible and acceptable in relation to the MCHH (*P*<.001).

mHealth technology interventions in maternal and child health are increasing worldwide [23]. There is a call for improvement in the content and quality of health care apps, including evidence-based information consistent with guidelines, supported and developed by health care providers with credible and reliable resources [24-26]. A study in 2015 from Australia found that only 40% of the apps involved health care professionals and provided evidence-based content, while 30% implemented user privacy security measures [24]. The KhunLook app was developed by health care providers and app developers in conjunction with parents since conception. It uses evidence-based content and honors user data privacy. Both themes from phase 1 support the development of a mobile app to assist with child health supervision.

Mobile apps are used to support interventions for maternal and child health care [27,28]. Studies have used mobile apps to connect and send data between health care providers and patients or use it for means of notifications, with positive outcomes [26-34]. In Kenya, the IFA app was found to shift the relationship between the caregiver and health care provider from feeling harassed for data to being genuinely interested [33]. For child health supervision in Thailand, mobile phones were successfully used to connect with parents for child immunization appointments via text messages, collect data via image capture, and conduct hearing screenings [29,30,34]. Findings from phase 2 add to the benefit of using a mobile app to ease the process of assessing growth parameters and instantly taking numbers to a deeper meaning of growth status. We found that 91.2%-97.1% of the parents who used the app could correctly assess their child’s growth parameters, whereas only 86.4%-95.5% of the parents who used the MCHH could do so. Thus, our findings suggest that the KhunLook app yields the benefits of mHealth services and can support parents in assessing their child growth similar to the MCHH.

Successful benefits of mHealth services require acceptability and engagement from the user [23,35]. A study found that use of a mHealth intervention in patients with type 2 diabetes is associated with its acceptability [35]. According to our findings, KhunLook was well accepted. Results from a web-based survey rated the app to be feasible for parents for initial assessments and easier to use than the MCHH. Parents provided a higher mean score for the KhunLook app than the MCHH with statistical significance (*P*<.001). Most of the parents (317/356, 89.0%) preferred to use the mobile app rather than the MCHH, 93.5% (333/356) stated that they would continue to use the app and 96.9% (345/356) would recommend others to use the app. Our findings support the KhunLook app as an acceptable mode of delivery for child health supervision for parents.

We developed the KhunLook app based on the belief that parental involvement would increase early detection and adequate childcare. Initially, the goal was to develop the app to compliment the MCHH. During the process, it has become eminent that the maintenance of content update, feature development, and user service is crucial. However, for the app to reach its potential, implementation at a wide scale is imperative. We started with a small team of health care providers, app developers, and users. Later on, we involved stakeholders on a wider scale. While drafting this paper (August 2020), the KhunLook app has been downloaded more than 320,000 times. It is endorsed by the Ministry of Public Health, and a link to download the app is currently provided in the MCHH.

### Limitations

The results of this study are interpretations from a certain time and do not reflect the current app. The development of “KhunLook” in phase 1 and 2 involved a convenient sample of parents at a university hospital. Most of the parents were well educated and were willing to try new technology but the number of participants was too small to draw generalized conclusions.

### Conclusions

In this study, KhunLook, a Thai mobile app for child health supervision, was developed, validated for growth assessments, and was found to be well accepted for ease-of-use by parents. The full potential of this mHealth app is yet to be defined. Further studies on parental and clinical use should be conducted such as a randomized study involving a wider scale of users or studies to evaluate its impact on health behaviors and health outcomes.

## Abbreviations

MCHH: maternal and child health handbook
mHealth: mobile health
